# Research workshop to research work: initial steps in establishing health research systems on Malaita, Solomon Islands

**DOI:** 10.1186/1478-4505-8-33

**Published:** 2010-10-31

**Authors:** Michelle L Redman-MacLaren, David J MacLaren, Janella Solomon, Alwin Muse, Rowena Asugeni, Humpress Harrington, Esau Kekuabata, Richard Speare, Alan R Clough

**Affiliations:** 1 School of Public Health, Tropical Medicine and Rehabilitation Sciences, James Cook University, McGregor Rd, Smithfield, Cairns, Australia; 2 Kilu'ufi Hospital, Auki, Malaita, Solomon Islands; 3 Atoifi College of Nursing, East Kwaio, Malaita, Solomon Islands; 4 Atoifi Adventist Hospital, East Kwaio, Malaita, Solomon Islands; 5 Chief, East Kwaio, Malaita, Solomon Islands; 6 School of Public Health, Tropical Medicine and Rehabilitation Sciences, James Cook University, James Cook Drive, Douglas, Townsville, Australia; 7 School of Indigenous Australian Studies, McGregor Rd, Smithfield, Cairns, James Cook University, Australia

## Abstract

**Introduction:**

Atoifi Adventist Hospital is a 90 bed general hospital in East Kwaio, Malaita, Solomon Islands providing services to the population of subsistence villagers of the region. Health professionals at the hospital and attached College of Nursing have considerable human capacity and willingness to undertake health research. However they are constrained by limited research experience, training opportunities, research systems, physical infrastructure and access to resources. This brief commentary describes an 'Introduction to Health Research' workshop delivered at Atoifi Adventist Hospital in September 2009 and efforts to move from 'research workshop' to 'research work'.

**The Approach:**

Using a participatory-action research approach underpinned by decolonising methodologies, staff from Atoifi Adventist Hospital and James Cook University (Queensland, Australia) collaboratively designed, implemented and evaluated a health research workshop. Basic health research principles and methods were presented using active learning methodologies. Following the workshop, Atoifi Adventist Hospital and Atoifi College of Nursing staff, other professionals and community members reported an increased awareness and understanding of health research. The formation of a local Research Committee, improved ethics review procedures and the identification of local research mentors followed the week long workshop. The workshop has acted as a catalyst for research activity, increasing structural and human resource capacity for local health professionals and community leaders to engage in research.

**Discussion and Conclusions:**

Participants from a variety of educational backgrounds participated in, and received benefit from, a responsive, culturally and linguistically accessible health research workshop. Improving health research systems at a remote hospital and aligning these with local and national research agendas is establishing a base to strengthen public health research and practice on Malaita, Solomon Islands.

## Introduction

Health research in a majority world nation such as Solomon Islands is very challenging[1]. In Solomon Islands, resource and system constraints limit opportunities to plan or undertake health research. Internationally, health researchers have been challenged to close the 'know-do' gap[2]and undertake research that improves health outcomes[3]. Here we report on a health research workshop conducted at a remote hospital in Solomon Islands to enhance local health research capacity. We also report on how lessons from the research workshop are informing future research.

Solomon Islands is an archipelago Pacific Island country of 595 000 people speaking over 70 languages[4,5] Over 80% of Solomon Islanders live a subsistence lifestyle in village settings and the country is ranked 135/182 on the UNDP Human Development Index[6]. Solomon Islands is re-establishing governance and civil systems following the breakdown of law and order from 1998 to 2003[7]. Public health priorities for Solomon Islands include rural water supply, improving health centres and addressing tuberculosis, malaria, HIV/AIDS and sexually transmitted infections and common childhood diseases[8].

Atoifi Adventist Hospital (AAH) is the largest non-government hospital in Solomon Islands. It is a 90 bed general hospital located in remote East Kwaio, Malaita Province. The hospital can be accessed by light aircraft, trading ship, motorised canoe and by foot. There is no road access. Most hospital employees live on the hospital campus including two Medical Officers and 27 Registered Nurses. Atoifi College of Nursing (ACON) is located on the AAH campus. ACON delivers a three year Diploma of Nursing to 60 students from across Solomon Islands and other Pacific Island countries. The hospital is located near the coast at Uru Harbour on the eastern side of the island of Malaita.

The hospital is located in an area of great ethic and religious diversity. Most people in the immediate coastal area live in large villages of up to 500 people and belong to one of five major Christian denominations. People in the nearby mountains live in small family hamlets and practice Ancestral religion. Many of people who practice Ancestral religion are unable to access hospital services because the entire hospital building is culturally taboo due to the maternity ward being located within the hospital building. This means it is not possible to enter the hospital without serious social, spiritual and cultural consequences requiring compensation to the ancestral spirits. After managing the medical laboratory at AAH 1992-1994, DM subsequently (2000 - 2006) used a participatory action research approach to collaboratively investigate barriers to health care at AAH and collective approaches to provide culturally appropriate health care for people who practice Ancestral religion. During this period enduring personal and professional relationships were established with many AAH employees and community leaders[9-12].

In September 2008, DM arranged for AAH Director of Nursing, AAH Mental Health Nurse and EK (a community chief) to travel to Cairns, Australia to deliver a presentation on incorporating spiritual paradigms into mental health services at AAH, from both an institutional and community perspective. While in Cairns these men met with DM and AC and discussed ways to build on collaborative health research opportunities and strengthen health research systems at AAH/ACON. Given leaders from AAH and ACON contribute to numerous professional associations and boards and make significant contributions to local, provincial and national health care delivery and policy forums, AAH and ACON are well positioned to contribute to local and national health research in Solomon Islands. An 'Introduction to Health Research' workshop was proposed and ultimately delivered at AAH/ACON in September 2009 following a formal invitation to JCU health researchers by AAH chief medical officer. This brief commentary outlines the workshop and describes initial steps taken to establish and strengthen health research systems at this remote hospital.

## The Approach

### The Workshop

In September 2009, a five day health research workshop was held at AAH involving 102 medical professionals, auxiliary staff, nursing students, local school teachers, chiefs and community leaders. Action research approaches underpinned by decolonising research methodologies informed the design and implementation of the workshop, including monitoring and evaluation processes[13,14]. Sessions were facilitated by three public health researchers from James Cook University; (AC, DM, MRM) two of whom (DM, MRM) had worked or conducted research at AAH previously, spoke Solomon Islands Pijin and had long-term relationships with many workshop participants.

Morning and evening sessions were facilitated each day. Sessions covered the same content but were facilitated in different locations on the AAH/ACON campus and used different languages. This allowed participants to choose the time, location and language of delivery and ensure equitable and culturally safe access for workshop participants. Two hour evening sessions were facilitated in the AAH chapel, used English and were primarily attended by nursing students, hospital staff and auxiliary workers. Two-hour morning sessions were facilitated in an ACON classroom and predominantly used Solomon Islands Pijin. Morning sessions were primarily attended by chiefs and community leaders. Some chiefs and community leaders from Christian villages were unable to attend the evening sessions because the use of English or the logistical challenge of travelling at night. Other chiefs, who practice Ancestral religion, were unable to enter the hospital building because of cultural taboos associated with entering a building used for childbirth[12]. Facilitating the workshop in two parallel sessions allowed participation from a variety of social, religious and professional backgrounds and ensured effective communication in a culturally safe location. Active learning methodologies were employed to allow participants to consider new ideas and try them out[15]. This was enacted by creating spaces for individual exercises and reflection, discussions in pairs and small group activities.

The workshop included: i) an overview of health research, ii) finding a research topic, iii) quantitative and qualitative research methods, and iv) writing and reporting research results. Research planning, methods and reporting were discussed using examples from facilitators' previous research in Solomon Islands and Australia. That research is underpinned by the 'worldview' of the researcher was explored. Other topics explored included: defining a research problem; conducting structured and semi-structured interviews; focus group discussion techniques. Participants engaged in practical exercises where qualitative data was collected, coded, analysed and discussed in groups.

Blank research proposal outlines were distributed at the beginning of the workshop with interested participants supported to develop brief research proposals as the workshop progressed. Research interests reflected the eclectic range of participants: TB treatment, neo-natal cord infections, HIV/AIDS prevention, oral thrush in children, respiratory infections, ethics processes, post-operation abscesses, reading skills in school children, nursing student participation in chapel, student absenteeism and hospital electricity demands.

### Monitoring and Evaluation

Monitoring and evaluation occurred throughout the workshop. At the completion of each session participants were invited to write a *One Minute Reflection*[16] on two questions:

(i) What was the most useful, meaningful or intriguing thing you learned during this session?

(ii) What question(s) remain uppermost in your mind as we end this session?

Responses were discussed between AAH/ACON workshop co-ordinators and JCU facilitators following each session. A verbal summary of *One Minute Reflections *was returned, discussed and elaborated on at the beginning of subsequent workshop sessions. Sessions were adapted to respond to *One Minute Reflection *replies. Between 56-89% of workshop participants completed *One Minute Reflections*. Responses were written in both English and Solomon Islands Pijin. At the completion of the workshop MRM summarised the 342 *One Minute Reflection *responses. DM, RA, HH and EK further reviewed and refined summaries with MRM (Table 1).

**Table 1:** Summary of One Minute Reflections

Topic	Participants written comments
Facilitation	Exercises really helped Thank you for examples of open-ended questions Practice seems to make us clearly understand what means by data analysis and collection Need more time to really understand this

Qualitative	Thanks for helping me understand open and closed ended questions Structured and semi-structured interview Understand what to observe during observation method in research Learnt about idea of triangulation Think this kind of research (qualitative) useful in Kwaio How do I structure these questions? How often will I have to do the observations and interviews to each place or people? I want to know more about coding data When our coding was presented to the front it made us think of what to say/write

Quantitative	Many ways can show data analysis e.g. tables, graphs etc How to analyse data (including calculations, %, gender) Problem here is Excel since have no idea how to use a computer Learn how to do quantitative research analysis using pen & paper-simple way Formulating tables

Research Infrastructure	How will I effectively conduct research despite difficulties with resources and financial difficulties? Who will assist me? Will I manage research by myself? How can I put this to practice since I have no money? This is because for so many years Form 7 leavers are doing nothing in the country since there are no scholarships?

Research Practice	The way to evidence-based practice How many types of theory are there? What is theory? Does the research done meet actual needs or problems at hand? Will research be the solution or part of it? World view (beliefs, ideas)

Research Skills	Been doing research but not systematically Literature review session-how to do a literature review Ways to analyse research and different structures of research Know how to collect quantitative & qualitative data I leant clearly what a research synopsis is How can I complete findings? How can I structure a research question? How will I collect data? Do I need to do both types of research? How can I put this research into a report? Still need to know how to present the information collected

Terminology	Good to learn terminology Definition of research terms: cross-sectional study, synopsis, coding, raw data Big terminology-needed more ground work Please explain the meaning 'hypothesis', 'scientific theory', 'theory', falsify

Workshop Resources	There should be handouts-that would make it easier to understand

**Figure 1:**
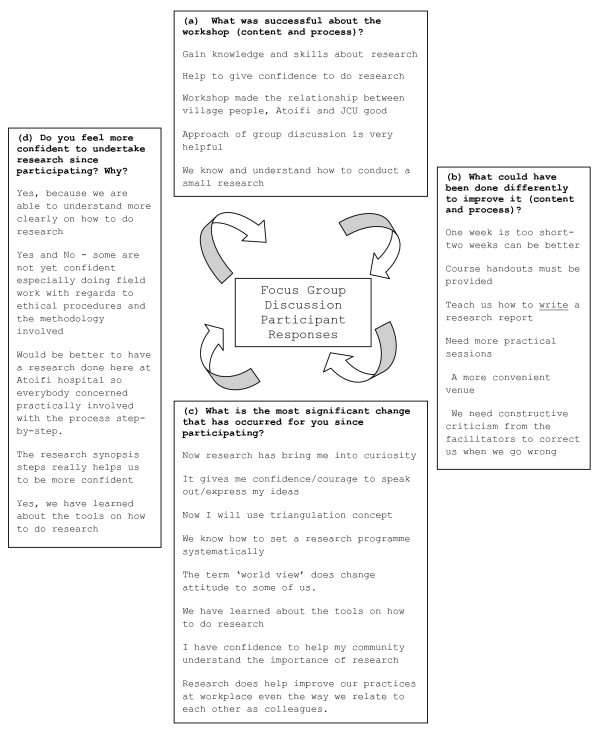
**Focus Group Discussion Participant Responses**.

On the final day of the workshop participants were asked to evaluate the content and process of the week long workshop in one of seven focus group discussions. Senior AAH/ACON staff and a chief facilitated the focus group discussions to consider: (a) What was successful about the workshop (content and process)?; (b) What could have been done differently to improve it (content and process)?; (c) What is the most significant change that has occurred for you since participating?; (d) Do you feel more confident to undertake research since participating? Why? Participant responses are summarised in Figure 1: Focus Group Discussion Participant Responses.

Seven weeks after the workshop MRM returned to AAH to collaboratively review monitoring and evaluation results with senior AAH/ACON staff and a chief, feedback workshop evaluation to workshop participants and collaboratively write the workshop report and draft publications. MRM, RA, HH and EK used Appreciative Inquiry methodology[17] to reflect upon the experience of the one-week workshop using a strengths approach and to build energy and research capacity for future research. Four questions were considered: 1. What 'gave life to the workshop? 2. What 'could have been'? 3. What 'should be'? 4. What is needed to 'sustain change'? Responses were collated and are summarised in Table 2: Appreciative Inquiry Reflection on Research Workshop.

**Table 2:** Appreciative Inquiry Reflection on Research Workshop

Question	Facilitators' Responses
What 'gave life' to the workshop?	Number and diversity of people Human capacity of participants Use of concrete, culturally accessible examples Overt interest in research by participants from increasingly educated backgrounds Desire to employ evidence-based practice Limited training opportunities at AAH/ACON meaning enthusiastic participation Historically, research had been perceived as something belonging to outsiders (white man) but the workshop demonstrated opportunities for Solomon Islanders to lead research.

What 'could have been'?	Further exploration of theoretical foundations and research systems (e.g. ethics) Smaller groups working on self identified research questions over a longer period

What 'should be'?	Local and international health research mentors supporting emerging researchers Functioning research systems at AAH/ACON with clear steps for researchers

What is needed to 'sustain change'?	Systems to support research at AAH Links with national and international health research systems

### Immediate Impact of Workshop

*"Now research has bring me into curiosity" - (*Workshop Participant)

The immediate impacts of the workshop (including emerging questions) were reported by participants in *One Minute Reflections*. One participant stated it was *"good to know the basics of research" *and it was *"very clear and simple"*, however *"I'd like to know more about how to analyse data." *Practical exercises stimulated further questions: *"I now appreciate interviewing and developing themes with the person/people interviewed" *but *"Can I make reactions while (asking) probing questions?"*

Focus group discussion participants reported an increase in knowledge about health research: *"Workshop participants do acquire basic knowledge about research and now able to do research synopsis. Generally the participants are happy and satisfied. This is because (a) use of simple language e.g. English to Pijin, (b) facilitators interacts more with participants." *Participants also reported a growing confidence to undertake research activities and motivation for further education: *"Me garem confidence for helpem community blo me for undertandim importance blo research" *(I have confidence to help my community understand the importance of research); *"Help to give confidence to do research and motivate us for further studies."*

### Ongoing Impact

In the week following the workshop a seven member research committee was established at AAH. Members of the committee include health professionals, a community high school principal and a community chief. The research committee plans to co-ordinate research activities at AAH/ACON and provide leadership for emerging local researchers. Research systems are also being strengthened at AAH through the enhancement of research ethics committee documentation and procedures. In February 2010 a proposal for a collaborative JCU-AAH HIV prevention study was submitted to a major funding body. In March 2010 three senior AAH/ACON staff were appointed to adjunct positions at the School of Public Health, Tropical Medicine and Rehabilitation Sciences at James Cook University. Two of these (RA and HH) travelled to Cairns, Australia in September 2010 to present the process and outcomes of the research workshop with MRM at the 2010 Fulbright Symposium[18]. This led to a further application for funding for a collaborative project to investigate a number of Neglected Tropical Diseases at AAH and surrounding communities.

## Discussion and Conclusions

Participants' eagerness to actively engage in the workshop conducted in a location where there are few educational opportunities encouraged a positive learning environment for participants from a range of social, cultural, religious and professional backgrounds. The pre-existing relationships between AAH/ACON and JCU researchers, the use of Solomon Islands Pijin, the involvement of community chiefs and the understanding and use of cultural constructs by facilitators all contributed to workshop success.

Limitations to the success of the workshop included considerable human and resource constraints. There is currently no dedicated health researcher at AAH/ACON, with research activity being conducted in addition to regular duties (e.g. Director of Nursing, Principal of ACON, Medical Officer). Infrastructure limitations include minimal electricity (at time of writing: eight hours per day), limited computing, printing, telephone and intermittent and slow internet access. Many library materials are outdated. Despite these limitations participants from a variety of educational backgrounds participated in, and received benefit from, a responsive, culturally and linguistically accessible health research workshop.

Using an action research approach underpinned by decolonising research methodologies allowed Australian and Solomon Islander colleagues to utilise each other's skills and knowledge to collaboratively design, implement, monitor and evaluate the workshop. Utilising methods which included different options of time, location and language of delivery contributed to a culturally safe learning environment. Active learning methodologies allowed participants to build upon existing knowledges and more actively participate. The *One Minute Reflections *allowed rapid monitoring of each session and provided opportunities to modify subsequent sessions in response to participant's concerns. Having the workshop participants evaluate the workshop utilising skills learned during the workshop (focus group discussion and data coding) and reviewing the results using an appreciative inquiry methodology re-enforced the utility of skills acquired or strengthened during the workshop.

Collaboratively writing this publication and RA, HH and MRM presenting at an international symposium demonstrates the ongoing commitment to collaborative partnership. This paper is a description of an action research approach for a health research workshop at a remote Solomon Islands hospital. However, broader principals of: responding to an issue of concern from within the community; collaboration; honouring multiple knowledges; respect of difference; building on strengths; and flexibility are all relevant for conducting action research in other resource challenged, spiritually diverse and/or post-colonial contexts[19-21].

Participants have requested a follow-up workshop to cover a more comprehensive range of topics and to build on the successes of the initial workshop. A regular review of research activities and research infrastructure at AAH could monitor changing capacity and resource needs and collaborative research successes. Strengthening collaborative relationships between AAH, JCU, Ministry of Health and Medical Services (MHMS), Solomon Islands Institute of Medical, Training and Research Institute (SIMTRI) or other research institutions could create further opportunities to enhance health research capacity with the aim to improve health outcomes for people in East Kwaio and Solomon Islands. Ongoing support and collaboration is required at this remote Solomon Islands hospital to ensure initial steps to strengthen health research systems can align with local and national research agendas and ensure progress from 'research workshop' to 'research work'.

## Competing interests

The authors declare that they have no competing interests.

## Authors' contributions

MRM co-facilitated the design and implementation of the research workshop, led the collection and analysis of monitoring and evaluation data, drafted and edited the manuscript. DM co-facilitated the design and implementation of the research workshop, collected monitoring and evaluation data, critically reviewed data analysis and edited the manuscript. JS, AM, RA, HH and EK facilitated FGDs, collected evaluation data, reviewed qualitative data analysis and edited the manuscript. RS co-facilitated the design of the workshop and edited the manuscript. AC co-facilitated the design and implementation of the research workshop and edited the manuscript. All authors read and approved the final manuscript.

## References

[B1] World Health Organisation (WHO) National Health Research Systems:report of an international workshop. Cha-am, Thailand 12-15 March 2001 2002 Geneva: World Health Organisation

[B2] Momen H Lessons from the Field Bulletin of the World Health Organization 2005 83 2 2

[B3] Sanson-Fisher RW Campbell EM Htun AT Bailey LJ Millar CJ We are what we do: research outputs of public health Am J Prev Med 2008 35 380 385 10.1016/j.amepre.2008.06.039 18687567

[B4] The World Factbook-Solomon Islands https://www.cia.gov/library/publications/the-world-factbook/geos/bp.html

[B5] Tryon D Hackman B The Languages of the Solomon Islands: An Internal Classification 1983 Canberra: Pacific Linguistics C-77

[B6] United Nations Development Program Human Development Report 2009: Solomon Islands 2009 UNDP

[B7] Moore C Happy isles in crisis: the historical causes for a failing state in Solomon Islands, 1998-2004. Asia Pacific Pr 2005

[B8] SPC Strategic Engagement PaPF Solomon Islands Country Profile 2008 Noumea: Secretariat of Pacific Community

[B9] MacLaren D Kastom and Health: a study of indigenous concepts of custom, health and and appropriate health care within Kwaio, Malaita, Solomon Islands 2000 Griffith University, School of Public Health

[B10] MacLaren D Culturally Appropriate Health Care in Kwaio Solomon Islands: An Action Research Approach 2006 Griffith University, School of Public Health Group

[B11] MacLaren D Asugeni J Asugeni R Kekeubata E Incorporating sociocultural beliefs in mental health services in Kwaio, Solomon Islands Australas Psychiatry 2009 17 Suppl 1 S125 127 10.1080/10398560902948381 19579125

[B12] MacLaren D Kekeubata E Reorienting health services through community health promotion in Kwaio, Solomon Islands Promot Educ 2007 14 78 79 10.1177/10253823070140021701 17665704

[B13] Stringer E Genat WJ Action research in health CJNR 2004 36 147 151

[B14] Denzin NK Lincoln YS Smith LT (Eds) Handbook of Critical and Indigenous Methodologies 2008 Thousand Oaks: Sage

[B15] Silberman M Active learning: 101 strategies to teach any subject 1996 Boston: Allyn and Bacon

[B16] Davis BG Tools for Teaching 1993 San Fransisco: Jossey-Bass Publishers

[B17] Cooperrider DL Whitney D Stavros JM Appreciative Inquiry Handbook: The first in a Series of AI Workbooks for Leaders of Change 2003 Bedford Heights: Lakeshore Communications, Inc

[B18] Redman-MacLaren M Asugeni R Harrington H Research Workshop to Research Work: working together to develop research skills at Atoifi Adventist Hospital, Solomon Islands Sustainable Societies in the Tropical World: 2010 Fulbright 60th Anniversary Symposium. Cairns, Australia 2010

[B19] Lykes MB Mallona A Reason P, Bradbury H Towards Transformational Liberation: Participatory and Action Research Praxis Handbook of Action Research 2008 Thousand Oaks: Sage

[B20] Stringer E Action Research 2007 3 Thousand Oaks: Sage

[B21] Wadsworth Y 'Beloved Bangladesh': A western glimpse of participatory action research and the animator-resource work of Research Initiatives Bangladesh Action Research 2005 3 417 435 10.1177/1476750305058490

